# Pathogenesis of *Plasmodium berghei* ANKA infection in the gerbil (*Meriones unguiculatus*) as an experimental model for severe malaria

**DOI:** 10.1051/parasite/2017040

**Published:** 2017-10-16

**Authors:** Quazim Olawale Junaid, Loke Tim Khaw, Rohela Mahmud, Kien Chai Ong, Yee Ling Lau, Prajakta Uttam Borade, Jonathan Wee Kent Liew, Sinnadurai Sivanandam, Kum Thong Wong, Indra Vythilingam

**Affiliations:** 1 Department of Parasitology, Faculty of Medicine, University of Malaya, Lembah Pantai, 50603 Kuala Lumpur Malaysia; 2 Department of Biomedical Science, Faculty of Medicine, University of Malaya, Lembah Pantai, 50603 Kuala Lumpur Malaysia; 3 Department of Pathology, Faculty of Medicine, University of Malaya, Lembah Pantai, 50603 Kuala Lumpur Malaysia; 4 Department of Biological Science, Faculty of Science, Federal University Kashere, Gombe State Nigeria; 5 Department of Pathology, School of Medicine, International Medical University, 57000 Kuala Lumpur Malaysia

**Keywords:** Gerbil, *Plasmodium berghei* ANKA, Severe Malaria, Pathogenesis, Cytokines, *In situ* hybridization

## Abstract

*Background*: As the quest to eradicate malaria continues, there remains a need to gain further understanding of the disease, particularly with regard to pathogenesis. This is facilitated, apart from *in vitro* and clinical studies, mainly via *in vivo* mouse model studies. However, there are few studies that have used gerbils (*Meriones unguiculatus*) as animal models. Thus, this study is aimed at characterizing the effects of *Plasmodium berghei* ANKA (PbA) infection in gerbils, as well as the underlying pathogenesis. *Methods*: Gerbils, 5-7 weeks old were infected by PbA via intraperitoneal injection of 1 × 10^6^ (0.2 mL) infected red blood cells. Parasitemia, weight gain/loss, hemoglobin concentration, red blood cell count and body temperature changes in both control and infected groups were monitored over a duration of 13 days. RNA was extracted from the brain, spleen and whole blood to assess the immune response to PbA infection. Organs including the brain, spleen, heart, liver, kidneys and lungs were removed aseptically for histopathology. *Results*: Gerbils were susceptible to PbA infection, showing significant decreases in the hemoglobin concentration, RBC counts, body weights and body temperature, over the course of the infection. There were no neurological signs observed. Both pro-inflammatory (IFNγ and TNF) and anti-inflammatory (IL-10) cytokines were significantly elevated. Splenomegaly and hepatomegaly were also observed. PbA parasitized RBCs were observed in the organs, using routine light microscopy and *in situ* hybridization. *Conclusion*: Gerbils may serve as a good model for severe malaria to further understand its pathogenesis.

## Abstract (fr)

*Contexte :* Alors que la quête de l'éradication du paludisme se poursuit, il faut encore mieux comprendre la maladie, en particulier en ce qui concerne sa pathogenèse. Ceci est facilité, outre les études *in vitro* et cliniques, principalement par des études de modèles souris *in vivo*. Cependant, il existe peu d'études qui ont utilisé des gerbilles (*Meriones unguiculatus*) comme modèles animaux. Ainsi, cette étude vise à caractériser les effets de l'infection par *Plasmodium berghei* ANKA (PbA) chez les gerbilles, ainsi que la pathogenèse sous-jacente. *Méthodes :* Les gerbilles, âgées de 5 à 7 semaines, ont été infectées par PbA par injection intrapéritonéale de 1 × 10^6^ (0,2 mL) de globules rouges infectés. Le parasitisme, le gain/perte de poids, la concentration d'hémoglobine, le nombre de globules rouges et les changements de température corporelle dans les groupes témoins et infectés ont été surveillés sur une durée de 13 jours. L'ARN a été extrait du cerveau, de la rate et du sang total pour évaluer la réponse immunitaire à l'infection à PbA. Les organes, y compris le cerveau, la rate, le cœur, le foie, les reins et les poumons ont été extraits de manière aseptique pour l'histopathologie. *Résultats :* Les gerbilles étaient sensibles à l'infection à PbA, ce qui a entraîné une diminution significative de la concentration d'hémoglobine, du nombre de globules rouges, des poids corporels et de la température corporelle au cours de l'infection. Aucun signe neurologique n'a été observé. Les cytokines pro-inflammatoires (IFNγ et TNF) et anti-inflammatoires (IL-10) étaient significativement élevées. La splénomégalie et l'hépatomégalie ont également été observées. Des globules rouges parasités par PbA ont été observés dans les organes, en utilisant la microscopie optique de routine et une hybridation *in situ*. *Conclusion :* Les gerbilles peuvent être un bon modèle pour le paludisme sévère et sa pathogenèse.

## Introduction

According to the World Health Organization (WHO), an estimated 214 million new cases of malaria and 438,000 deaths were recorded in the year 2015 [76]. To reduce this threat, there is still a need to better understand the underlying processes that result in severe disease outcome and mortality. One of the ways this can be achieved is by exploring different experimental models for malaria.

As in human malaria infections, rodent *Plasmodium* parasites vary in virulence depending on the species of *Plasmodium* and species of rodents or strains of mice [63,68]. Variation in *Plasmodium* parasite virulence can be explained with the suggestion that the clonal composition of the *Plasmodium* parasite may have an effect on the disease outcome; also, this can be regulated by mouse genetic background and the interplay dynamics between the clones and their hosts [2].

Severe malaria anemia (SMA) is a common occurrence in malaria endemic communities and is considered to be responsible for high morbidity and mortality in young children and pregnant women [35,46]. Previously, the clinical features and pathogenesis of severe malaria were attributed to either severe anemia due to destruction of red blood cells (RBC) or cerebral malaria (CM), which is caused by obstruction of small vessels of the brain by sequestered parasites [50]. However, the host has evolved a mechanism in controlling the degree of RBC destruction, which is beneficial to some, while detrimental to others.

The ANKA strain of *Plasmodium berghei* (PbA) has long been used as a model for experimental cerebral malaria (ECM) due to its high degree of reproducibility and the development of histopathological and neurological symptoms similar to human cerebral malaria (CM) [22,48]. A previous study by Bopp *et al.* [10] has shown that different mouse strains infected with PbA are either susceptible or resistant to ECM to varying degrees.

Early secretion of pro-inflammatory T-helper 1 (Th 1) cytokines is important in successful resolution of malaria infection through killing of parasites by macrophages, thus preventing immune-mediated damage [42]. Although pro-inflammatory cytokines are crucial in the clearance of *Plasmodium* parasites, their overproduction has been implicated in the symptoms that accompany *Plasmodium* infection [5,77]. On the other hand, the inability of the host to mount an effective pro-inflammatory response, may instead lead to unrestricted parasite replication, thus contributing to severe immunopathology [15]. These observations suggest that the balance between pro-inflammatory and regulatory immune responses during malaria infection is an important factor in determining the disease outcome.

Gerbils have been used in various areas of biomedical research, such as stroke, behavior, parasitology, epilepsy, radiobiology, hearing and infectious disease research [39]. More importantly, gerbils have been established as a good experimental model for filarial nematodes [59,61], *Helicobacter pylori*-induced gastritis [8,78], and inflammatory bowel disease [9]. Although mice and rats have been used extensively for experimental malaria studies, there are still doubts concerning the extrapolation of findings to severe malaria in humans [48]. As a result, studying a relatively unexplored experimental model subjected to severe malaria will enhance our understanding of the disease. Moreover, studies involving *P. berghei* in gerbils are not recent [66,67,73], with the most recent study having been conducted over four decades ago [72].

The pathology of PbA infection in mice has been associated with accumulation of infected RBCs in the brain, but it is not clear whether cyto-adherence of PbA occurs in the microvasculature of the brain [16]. Baptista *et al.* [7] have demonstrated that parasitized RBCs together with CD8^+^ T cells play a crucial role in the onset of neuropathology in ECM. However, previous studies on PbA-mice models have also shown the presence and accumulation of iRBCs in various organs such as the brain, heart, liver, spleen, lungs and kidneys [14,23–25]. In addition, different methods and approaches have previously been used to identify and determine the accumulation of *Plasmodium* parasites in different organs [23,30,32,33].

In this study, the effect of PbA on gerbils was characterized focussing on immune responses.

## Material and methods

### Ethics approval

The protocol was approved by the Faculty of Medicine Institutional Animal Care and Use Committee (FOM IACUC), University of Malaya, Malaysia (2014/PARA/R/JOQ).

### Gerbils

Mongolian gerbils (*Meriones unguiculatus*), purchased from Charles River (USA) at approximately 4 wks old, were maintained and allowed to breed at the animal facility of the University of Malaya. Gerbils were maintained in individually ventilated cages and supplied with sterilized food and water *ad libitum*. About 74 gerbils of age 6-8 wks were used in all experiments, in accordance with institutional guidelines for animal care. All animals were handled humanely to minimize pain.

### Parasite and Infection

The *Plasmodium berghei* strain ANKA (MRA-311) parasite was obtained from the Malaria Research and Reference Reagent Resource Center (MR4, USA) and inoculated in gerbils via the intraperitoneal (ip) route. Briefly, frozen PbA parasitized red blood cells (pRBCs) were allowed to thaw at 37 °C for 3-5 mins, and 0.2 mL was injected into an uninfected gerbil to initiate infection. Blood was collected by cardiac puncture from the donor gerbil on day 5-7 post-infection, and diluted appropriately with phosphate buffer saline (PBS, pH 7.4), before re-introduction into new gerbils. Control animals were given only phosphate buffer saline (PBS, pH 7.4). Concurrently, parasites were also stored in liquid nitrogen with 10% glycerol in Alsever's solution (2.33 g of glucose, 1 g of sodium citrate and 0.52 g of sodium chloride in 100 mL double distilled water).

### Parasitemia, survival rates and disease assessment

Gerbils were inoculated intraperitoneally with different concentrations (10^7^, 10^6^, 10^5^, 10^4^, 10^3^, 10^2^) of PbA to determine their susceptibility to PbA infections. Parasitemias were monitored every 48 hrs by thin blood smears prepared from tail pricks. These were then fixed in methanol and stained with 3% Giemsa (Sigma, USA) solution for 45 mins and allowed to dry before examining under a light microscope. Parasitemias were quantified as the percentage of pRBCs in at least five microscopic fields, each containing approximately 200-250 RBCs. To evaluate their survival rate, gerbils were monitored daily for a period of 30 days.

The body weight of gerbils was measured using an electronic balance (A&D, Japan), while body temperature in the animals was measured with a thermometer (Rossmax TG380, Switzerland) by placing the thermometer probe 0.5-1 cm into the mouth. Hemoglobin (Hb) concentrations were determined by Hemocue AB (Angelholm, Sweden). Briefly, 5-10 μL of blood from a tail prick was pipetted into the cuvette, which was inserted into Hemocue and readings were taken. Total red blood cell count was determined using a hemocytometer (Marienfeld, Germany) under a light microscope. This was done by collecting 5-10 μL of tail pricked blood into heparinized hematocrit-capillary tubes (Hirschmann, Germany). It was then diluted with phosphate buffered saline (PBS), and stained with trypan's blue (Sigma, USA). Gerbils were assessed daily for clinical symptoms such as ruffled hair, hunchback, coma, convulsion, paralysis and wobbly gait.

### RNA Extraction and cDNA preparation

The animals were first anesthetized with ketamine (Troy Laboratories, Australia) and xylazine (Santa Cruz Animal Health, USA) intraperitoneally at the dose of 50 mg/kg and 2 mg/kg, respectively [39]. Blood was then drained from the gerbil through cardiac puncture into EDTA tubes (BD, USA) on ice. Total RNA was extracted from the blood using a GF-1 Blood Total RNA Extraction kit (Vivantis, Malaysia). Simultaneously, about 50-100 mg of the brain and spleen were surgically removed and the tissues snapped frozen in liquid nitrogen before extracting their total RNA with a Pure link RNA Mini kit (Life technologies, USA), in accordance with the manufacturer's instructions. The quantity and quality of the RNA were then assessed with the NanoDrop 2000 spectrophotometers (Thermo Fisher Scientific, USA). Total RNA of 2 μg was then converted to cDNA with a Super Script IV first-strand synthesis kit (Thermo Fisher Scientific, USA). The cDNA was then used as a template for the RT-PCR.

### Primers and Probes

Gene-specific oligonucleotide primers and probes for gerbils IL-4 (Gen Bank L37779), IL-6, IL-10 (Gen Bank L37781), IFN-γ (Gen Bank L37782), TNF (Gen Bank AF171082.1) and GAPDH (Gen Bank AB040445.1), have been published previously [78] and were incorporated in the study. Both the primers and probes for the Taqman RT-PCR assay used were from Applied Biosystems (Life technologies, USA), and these were further modified with the probes labeled reporter dye 6-carboxyfluorescein (FAM) at 5′ and quencher minor groove binder (MGB) at 3′. The list of primers and their sequences are given in Table 1.

**Table 1:** Primers and Probes used in this study.

Gene	Sequence
GAPDH	Forward primer: 5′ −CAAGCCCATCACCATCTTCCA- 3′
	Reverse primer: 5′ −CGGTGGACTCCACAACATACTC- 3′
	Probe: 5′ −FAM-CCGCCAACATCAAATG-MGB- 3′

IL-4	Forward primer: 5′ −CAGGGTGCTCCGCAAATTT- 3′
	Reverse primer: 5′ −GACCCCGGAGTTGTTCTTCA- 3′
	Probe: 5′ −FAM-ACTTCCCACGAGAGGTG-MGB- 3′

IL-6	Forward primer: 5′ −AGGATCCAGGTCAAATAGTCTTTCC- 3′
	Reverse primer: 5′ −TTCCGTCTGTGACTCCAGTTTCT- 3′
	Probe: 5′ −FAM-CCCAACTTCCGAGGCG-MGB- 3′

IL-10	Forward primer: 5′ −CAAGGCAGCCTTGCAGAAG- 3′
	Reverse primer: 5′ −TCCAGCCAGTAAGATTAGGCAATA- 3′
	Probe: 5′ −FAM-CTCCATCATGCCCAGCT-MGB- 3′

IFN-γ	Forward primer: 5′ −TTGGGCCCTCTGACTTCGT- 3′
	Reverse primer: 5′ −CAGTGTGTAGCGTTCATGGTCTCT- 3′
	Probe: 5′ −FAM-CCGGACTTGCCCTGC-MGB- 3′

TNF	Forward primer: 5′ −CACTCAGGTCCTCTTCTCAGAAC- 3′
	Reverse primer: 5′ −TGGTGGTTGGGTACGACATG- 3′
	Probe: 5′ −FAM-CCAGCGACAAGCCTG-MGB- 3′

FAM: 6-carboxyfluorescein; MGB: Minor Groove Binder.

### Analysis of cytokines by real-time PCR

Taqman PCR reactions for cytokine mRNA and housekeeping GAPDH mRNA levels were performed using an Applied Biosystems StepOnePlus Real-Time PCR system (Life technologies, USA). Taqman reactions (20 μL) were performed in triplicate using Taqman Fast Advanced Master Mix (Life technologies, USA) according to the manufacturer's instructions. The qPCR was carried out with slight modification to the manufacturer's instructions as follows: incubation at 50 °C for 2 min; polymerase activation at 95 °C for 20 sec; and 40 cycles of PCR with denaturation at 95 °C, 3 sec and annealing/extension at 60 °C for 30 sec. Comparative standard curves were generated as a result, with the data being presented as the mean fold change of the cytokine mRNA relative to the level of GAPDH mRNA.

### Histopathology

The spleen and liver of gerbils were assessed morphologically. The spleen index was calculated as the ratio of spleen wet weight (g) to body weight (g) × 100 (Specht *et al*., 2010), whereas the liver index was calculated as the ratio of liver wet weight (g) to body weight (g) × 100.

The carcasses of the gerbils subjected to clinical assessment and survival tests were preserved for post mortem. The organs were removed and preserved in 10% buffered formalin. The tissues were processed using an automated tissue processor (Leica TP1020, USA) and then embedded in paraffin wax. About 3-5 sections (4 μm) were randomly cut for both hematoxylin and eosin (H and E) staining and *in situ* hybridization.

### *In situ* hybridization

The *in situ* hybridization was performed as described by Ong *et al.* [56], with some modifications. Briefly, 4 μm tissue sections were dewaxed, rehydrated and depigmented with 10% ammonium (70% alcohol, 10 mins). The sections were then pretreated with 0.1% pepsin (30 mins, 37 °C), followed by incubation at 95 °C (10 mins) and 42 °C (overnight) in standard hybridization buffer, together with 1 μL of *Plasmodium* probe (courtesy of Dr. Lau Yee Lin and Dr. Ong Kien Chai). The slides were then passed through washing and blocking, followed by incubation with anti-digoxigenin-AP Fab fragments (Roche) (1:2000) at 4 °C overnight. The slides were washed, followed by liquid permanent red chromogen (Dako) incubation (2 hrs, room temperature). The slides were then counter-stained with Mayer's hematoxylin and mounted with Faramount aqueous mounting medium (Dako).

### Statistical analysis

All data were analyzed using GraphPad prism 6. The distribution of the data was assessed by a Kolmogorov-Smirnov test for normality testing. Data that followed normal distribution were analyzed by one-way ANOVA with Tukey's multiple comparison post-hoc tests in cases of significant differences. All results are expressed as mean ± S.E.M. (Standard Error of Mean) and considered statistically significant when *p* < 0.05.

## Results

### Susceptibility of gerbils to PbA infection

**Figure 1:**
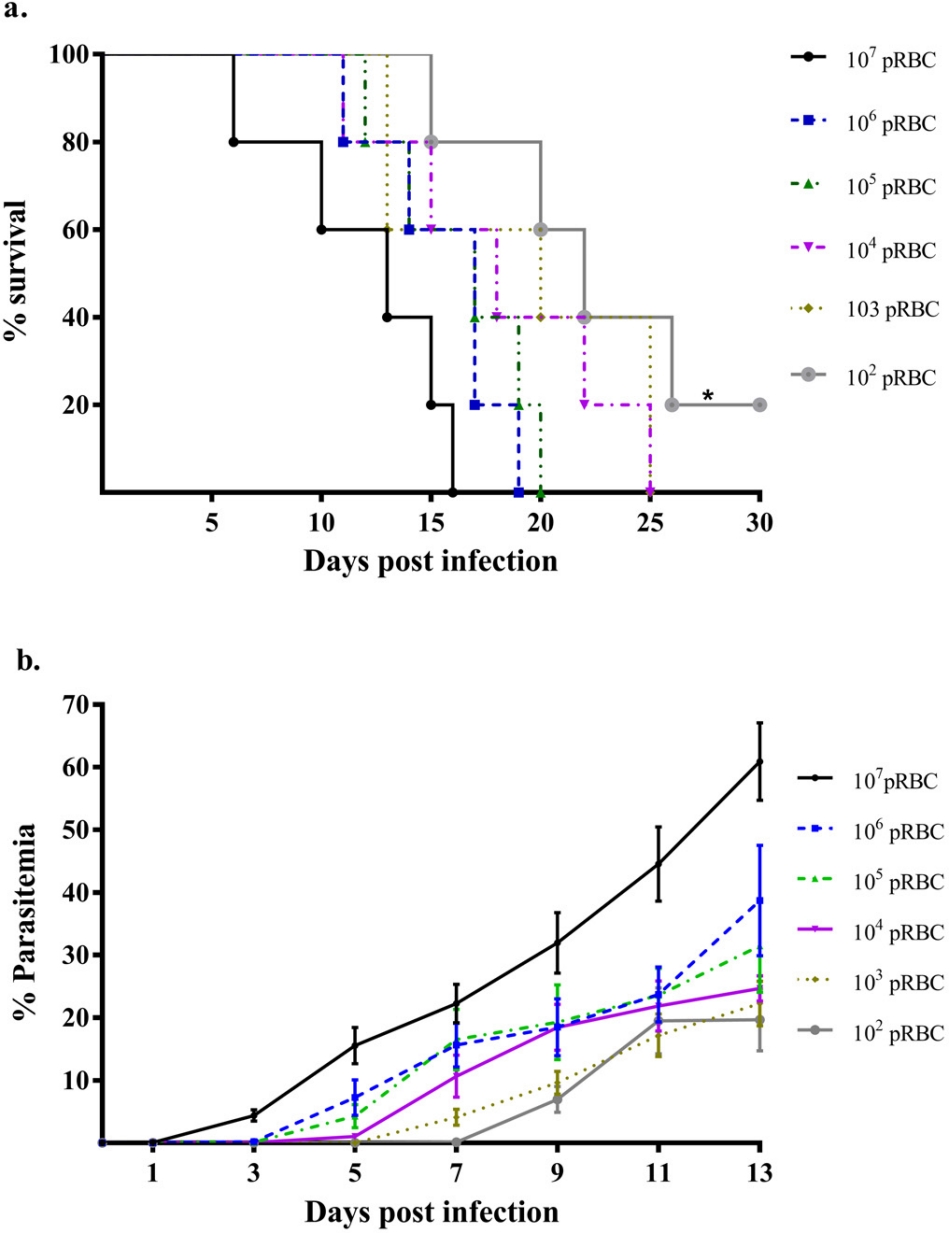
Susceptibility of gerbils to *P.* *berghei* ANKA (PbA) infection. Gerbils were infected intraperitoneally with different concentrations of PbA-infected red blood cells (pRBCs). Survival and parasitemia were monitored daily and every 2 days (respectively). a. Survival of gerbils according to the concentration of pRBC. b. Parasitemia level of PbA-infected gerbils according to the concentration of PbA, over 13 days post-infection. Lines represent mean ± S.E.M., N = 5 per group. A logrank (Mantel-cox) test was used to compare survival curves, *p < 0.05. One-way analysis of variance (ANOVA) was used to compare the differences in the level of parasitemia between the groups, *p* > 0.05.

Different concentrations (10^7^, 10^6^, 10^5^, 10^4^, 10^3^, 10^2^) of PbA pRBCs were administered intraperitoneally (ip), to determine the susceptibility of gerbils to PbA infections. Gerbils showed high susceptibility to PbA infections with 100% mortality recorded at all concentrations of pRBCs tested within 30 days post-infection (pi), with the exception of 10^2^ pRBCs, showing 80% mortality by day 27 pi (Figure 1a). However, the duration for which parasites were detected in gerbils (days post-infection) depended on the amount of PbA given. Overall, no later than day 5 pi, all gerbils tested positive. The parasitemia observed during the course of the experiment did not exceed 70% and there was no significant difference (F_(5, 42)_ = 1.579, *p* = 0.170) in the parasitemia level irrespective of pRBC concentrations (Figure 1b).

### Pathogenesis of PbA infection in gerbils

**Figure 2:**
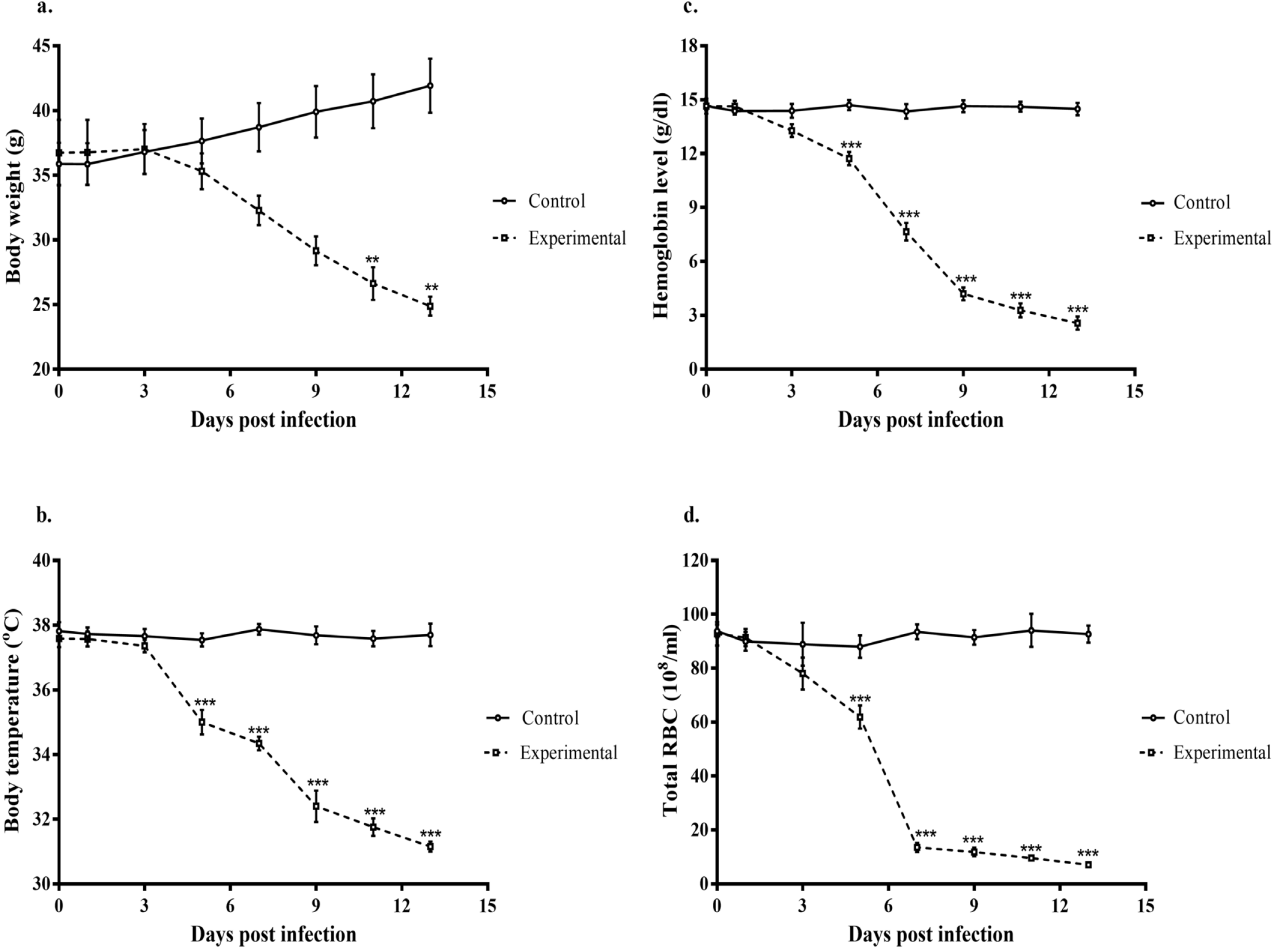
Body weight, body temperature, hemoglobin and total RBC during PbA infection. a. Body weight normalized to the body weight before infection (%). b. Body temperature as measured in infected and control group. c. Hemoglobin level as measured in infected and control group. d. Total RBCs as measured in infected and control group. Lines represent mean ± S.E.M., while N = 7 per group. All data are representative of three independent experiments and compared by one-way analysis of variance (ANOVA) with Tukey's multiple comparison post-hoc test for differences between groups (**p < 0.005; ***p < 0.0001). All data are representative of three different experiments.

A comparison between infected gerbils (1 × 10^6^ pRBCs in 200 μL of PBS) and the control group (200 μL of PBS only) was carried out to observe the possible pathological effects of PbA on the gerbil host. Both bodyweight and temperature changes during the course of infection were monitored. The body weight of PbA-infected gerbils was observed to decline from day 3 pi and showed a significant difference (F_(7, 48)_ = 8.328, *p* < 0.0001) at days 9 and 11 pi, while the control animals gained weight steadily (Figure 2a). However, the decline in body temperature observed during the course of infection was more evident from day 5 pi, and was highly significant (F_(7, 48)_ = 84.318, *p* < 0.0001) (Figure 2b).

The gerbils were also assessed for the level of anemia by quantifying the hemoglobin (Hb) level and total RBC counts during the course of PbA infection. It was observed that there was a significant (F_(7, 48)_ = 180.220, *p* < 0.0001) decline in Hb in the infected group compared to the control group over the time course (Figure 2c), and a similar pattern of decline was recorded in the total RBC count of the infected group (Figure 2d).

The common symptoms shared by the infected gerbils were ruffled hair and hunchback. However, none of the infected gerbils showed signs such as ataxia, convulsion and deviation of the head, except one gerbil that had partial paralysis (Table 2).

**Table 2:** Clinical symptoms observed in infected gerbils (n = 30).

Symptoms	Concentrations of pRBCs						Total N (%)
		
	10^7^	10^6^	10^5^	10^4^	10^3^	10^2^	
Ruffled hair	5	4	4	4	5	3	25 (83.3)
Hunchback	2	3	4	3	4	4	20 (66.7)
Ataxia	0	0	0	0	0	0	0 (0.0)
Convulsion	0	0	0	0	0	0	0 (0.0)
Wobbly gait	1	2	3	2	1	2	11 (36.7)
Deviation of the head	0	0	0	0	0	0	0 (0.0)
Paralysis	0	0	0	1	0	0	1 (3.3)*

Gerbils were observed daily to monitor their clinical symptoms. Five gerbils were assessed in each group.

* The gerbil showed partial paralysis of the hind limbs briefly before death. pRBCs: parasitized red blood cells.

### Cytokine response to PbA infection

Pro-inflammatory cytokines (such as IL-6, IFN-γ and TNF), anti-inflammatory cytokines (IL-4) and immunomodulatory cytokines (IL-10) were chosen to study the immune response of gerbils to PbA infection. The mRNA levels of these cytokines were quantified in the brain, spleen and blood of gerbils at various time points after intraperitoneal infection. Over all, IL-10, IL-6, IFN- γ and TNF were significantly increased in the brain and spleen, whereas in the blood, only IL-10 and IFN- γ were significantly elevated.

**Figure 3:**
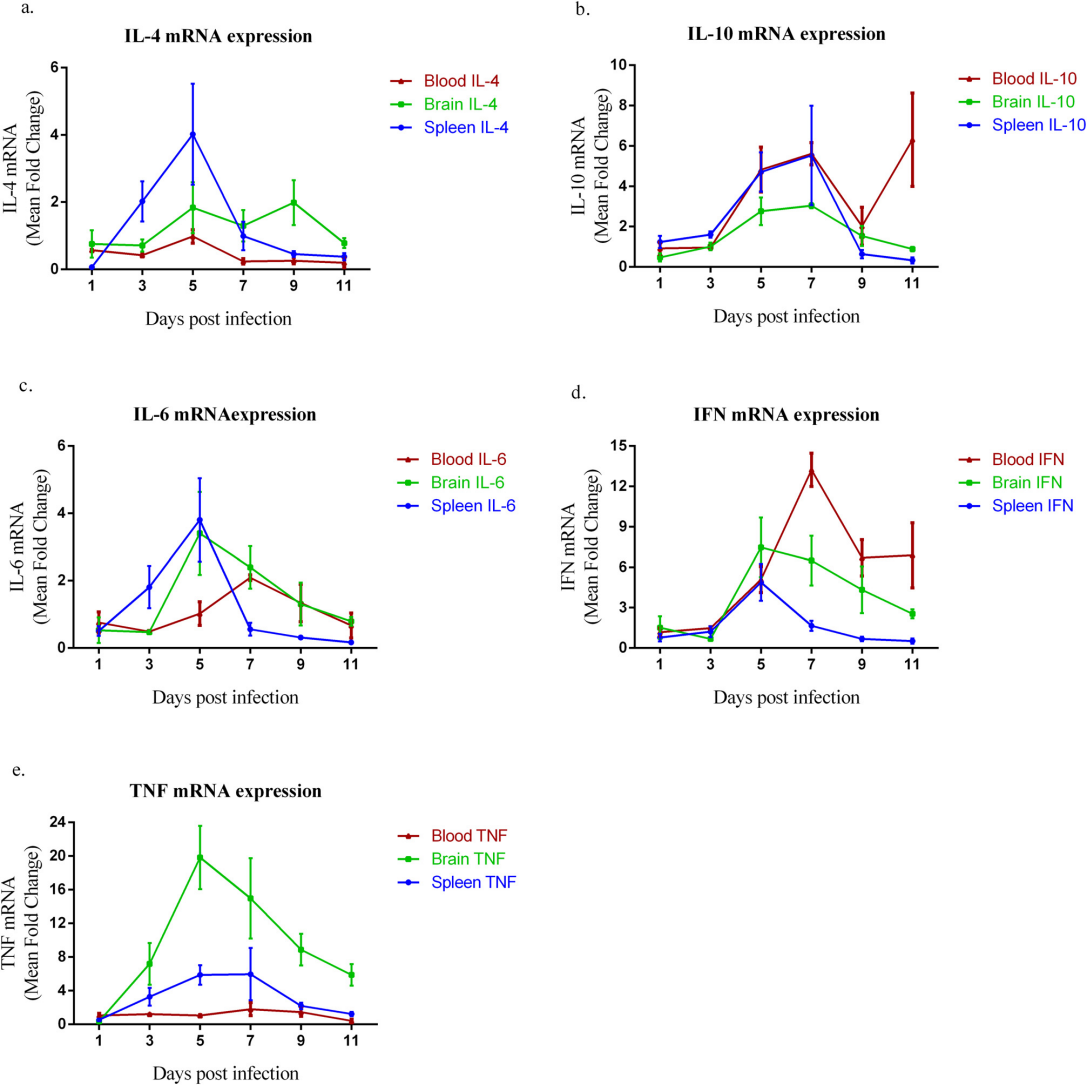
Quantitation of cytokine mRNA in the spleen, brain and blood. Gerbils were euthanized under anesthesia at days 1, 3, 5, 7, 9 and 11 after intraperitoneal inoculation with PbA. mRNA levels were measured by reverse transcription (RT)-PCR, and values were expressed as mean (±S.E.M., N = 5) fold changes compared with values from control, uninfected gerbils. a. IL-4 mRNA expression. b. IL-10 mRNA expression. c. IL-6 mRNA expression. d. IFN mRNA expression. e. TNF mRNA expression. All data are representative of two independent experiments and compared by one-way analysis of variance (ANOVA) with Tukey's multiple comparison post-hoc tests for differences between groups.

The IL-4 levels at days 3 and 5 post-infection (pi) in the spleen were more significantly elevated (F_(6, 28)_ = 4.511, *p* = 0.003) than at later time points, with a mean fold change of about 2.02 and 4.01, respectively. Although, there was a slight elevation of IL-4 in the brain at days 5 and 9 pi, it was not significant (F_(6, 28)_ = 1.185, *p* = 0.343) compared with other days pi. IL-4 was significantly (F_(6, 14)_ = 8.807, *p* = 0.000425) down-regulated throughout the time course in the blood, except day 5 pi which was at the same level as day 0 pi (Figure 3a).

The profile of IL-10, as expressed in all the tissues, showed consistent dual peaks at days 5 and 7 pi which was significantly (F_(6, 28)_ = 4.078, *p* = 0.005 in spleen; F_(6, 28)_ = 7.750, *p* < 0.0001 in brain) expressed (Figure 3b). However, IL-10 was most significantly (F_(6, 14)_ = 4.916, *p* = 0.007) elevated at day 11 pi in the blood with a 6.3 fold change (Figure 3b).

The level of IL-6 mRNA expressed in the spleen and brain at day 5 pi was significantly (F_(6, 28)_ = 5.493, *p* = 0.001; F_(6, 28)_ = 3.019, *p* = 0.021) up-regulated with 3.7 and 3.8 fold changes, respectively (Figure 3c). In the blood, IL-6 was only significantly (F_(6, 14)_ = 2.904, *p* = 0.047) expressed at day 7 pi (Figure 3c).

IFN in the spleen was significantly (F_(6, 28)_ = 6.784, *p* = 0.00016) elevated only at day 5 pi, with a mean fold change of 4.5, then sharply lowered until day 11 pi. However, there was a significant (F_(6, 28)_ = 3.910, *p* = 0.006; F_(6, 14)_ = 13.273, *p* < 0.0001) increase in the expression of IFN both in the brain and blood from day 5 to day 11 pi (Figure 3d).

The expression of TNF mRNA was significantly (F_(6, 28)_ = 2.924, *p* = 0.024) elevated from day 3 to day 9 pi in the spleen (Figure 3e). Although TNF mRNA was consistently high from day 3 to day 11 pi in the brain, the highest fold increase was observed on day 5 pi, with a fold change of 19.8, which was significantly (F_(6, 28)_ = 7.145, *p* = 0.00011) higher than expression at other time points. There was no significant difference (F_(6, 14)_ = 1.120, *p* = 0.399) in the expression of TNF in the blood throughout the time course (Figure 3e).

### Histopathology of PbA sequestration in the tissues

**Figure 4:**
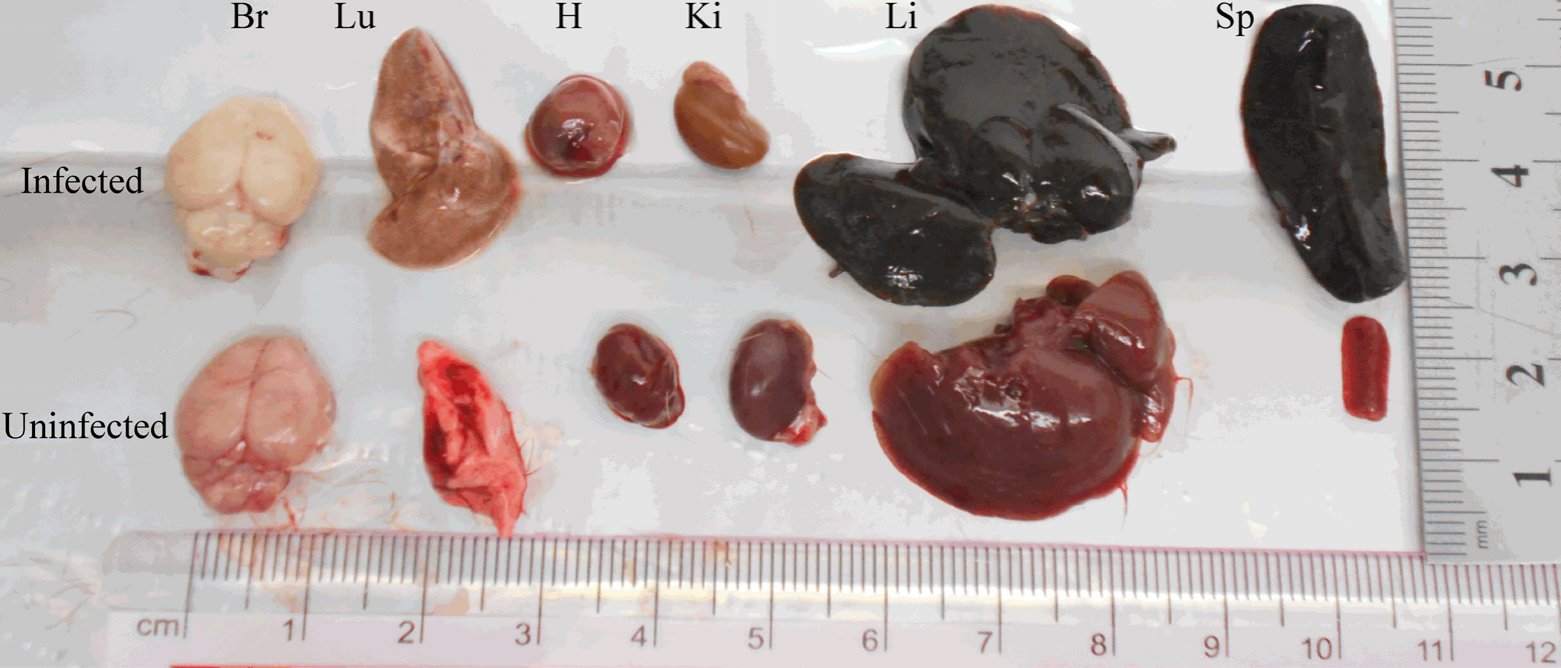
Morphology of organs harvested from gerbils at day 11 pi from infected and uninfected gerbils. The organs are: Br: Brain; Lu: Lungs; H: Heart; Ki: Kidney; Li: Liver; and Sp: Spleen. The liver and spleen are pigmented and enlarged (hepatomegaly and splenomegaly, respectively), while the infected kidney, lungs and brain are pale.

**Figure 5:**
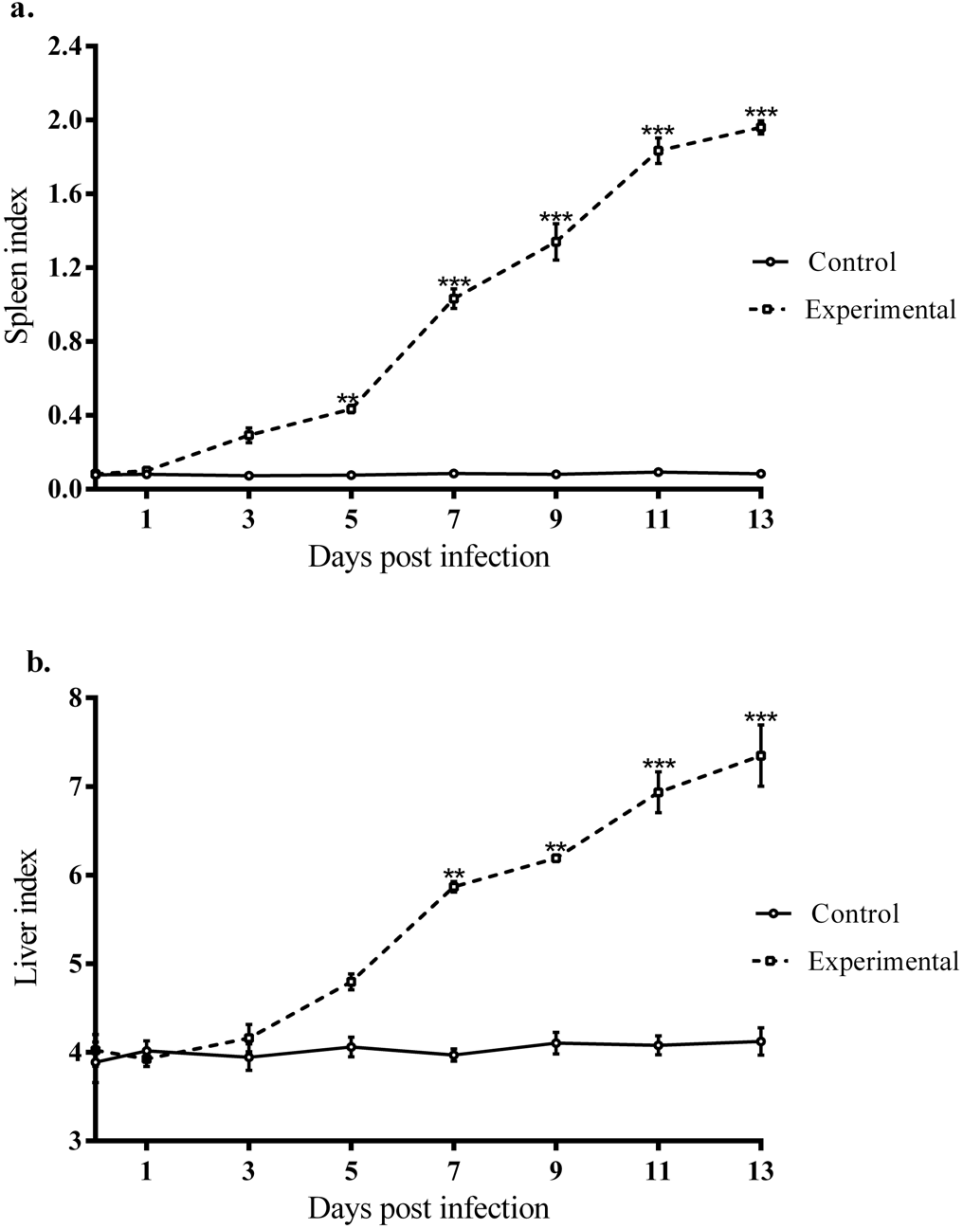
Spleen index and liver index (measured as the ratio of the organ's wet weight (g) versus body weight (g) × 100) over the 13-day time course. a. spleen index. b. liver index. Lines represent mean ± S.E.M., while N = 5. All data were compared by one-way ANOVA with Tukey's multiple comparison post-hoc tests for differences between groups (**p < 0.005; ***p < 0.0001).

The physical features of organs such as the brain, lungs, heart, kidneys, spleen and liver were examined. Among the anomalies observed in the organs of infected gerbils, were the enlargement and discoloration (pigmentation) of the spleen and liver compared to those in the control group (Figure 4). Quantitatively, there was an increase in the spleen indices (ratios of spleen wet weight (g) versus body weight (g) × 100) and liver indices (ratios of liver wet weight (g) versus body weight (g) × 100) compared to that of the control group. The increments in the spleen and liver indices were significant (F_(7, 32)_ = 224.205, *p* < 0.0001; F_(7, 32)_ = 59.919, *p* < 0.0001, respectively) from day 7 pi (Figure 5a and b).

**Figure 6:**
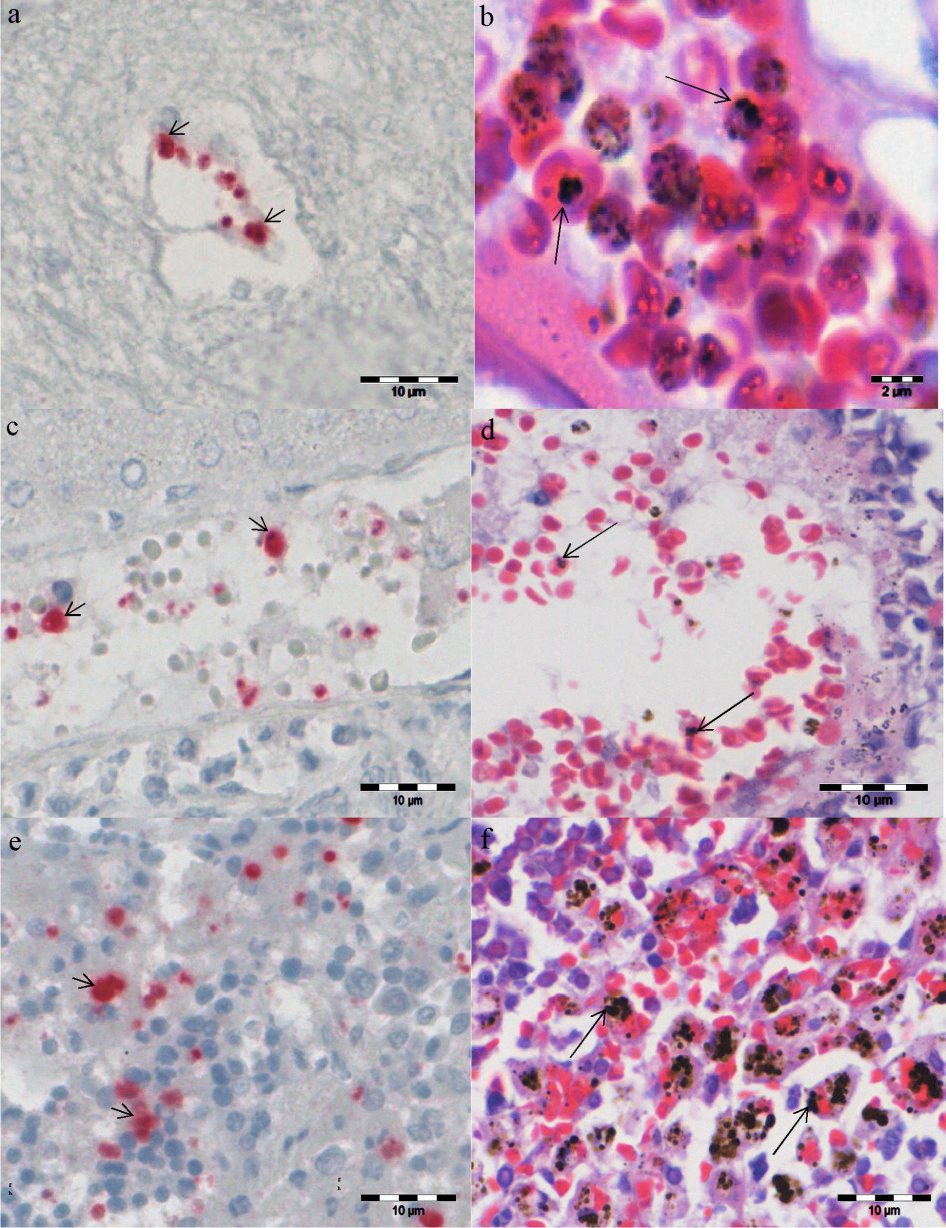
Histopathology of selected organs from infected gerbils. a, c, and e are representatives of *Plasmodium* probe *in situ* hybridization on infected brain, liver and spleen sections with depigmentation respectively. b, d, and f are representative of hematoxylin and eosin staining on infected brain, liver, and spleen sections without depigmentation, respectively. Short thick arrows show PbA-infected RBCs while long thin arrows show *Plasmodium* pigments (hemozoin). *Plasmodium* pigments (hemozoin) were removed with 10% ammonium oxide in 70% ethanol after de-paraffin on *in situ* hybridization slides. Post mortem was performed on 19 gerbils used for clinical assessment and survival tests.

Formalin fixed paraffin embedded (FFPE) tissues from both PbA-infected and uninfected gerbils were assessed on the following tissues: brain, kidneys, liver, lungs and spleen. The conventional method of hematoxylin and eosin (H and E) staining and *Plasmodium* genus DIG-labelled UTP probe *in situ* hybridization (ISH) on the tissues were compared. Results showed that PbA pRBCs were found in blood vessels of the tested organs and the ISH genus probe was sensitive and specific to the parasite (Figure 6).

## Discussion

The study of immunology in malaria and its underlying pathogenesis have been studied extensively, with the experimental models mostly focusing on combinations between different mouse strains and *Plasmodium species*. These studies have led to significant findings that have included identifying genes responsible for enhanced malaria survival in wide analysis of different mouse line genomes [10], recognizing mechanisms associated with strain-specific malaria infection [77], and determining the genotypic diversity of rodent malaria parasites [12,36,58].

However, the use of gerbils (*Meriones unguiculatus*) in parasitic infection studies has so far been limited mostly to *Brugia malayi*, and *B. pahangi* filarial parasites [52,59]. A previous study examined the infectivity and immunogenicity of mouse-adapted strains of *P. berghei* K173 on gerbils in the 1970s [72]. Previously, *P. berghei* K173 was described as causing non-cerebral malaria and death from other malaria-related complications in different mouse strains, unlike *P. berghei* ANKA which is more lethal and causes cerebral malaria [53,55]. Here, our study uses gerbil adapted to *P. berghei* ANKA (PbA) to study its effects on an immunological basis and the underlying pathology. The study showed that PbA infection causes severe malaria in gerbils in terms of body weight loss, lowered hemoglobin concentrations and RBC counts, as well as pigmentation and enlargement of the spleen and liver.

Many factors influence the clinical outcome of malaria infection in both humans and rodents. It had been suggested that infections caused by malaria parasites can vary in virulence depending on the complexity between environmental factors and the host, as well as parasite genetics [63,68]. The present study showed gerbils to be highly susceptible to PbA infection, even at low dosages (10^2^ and 10^3^ pRBC) of PbA. The susceptibility of different mouse strains to PbA infection had been characterized previously [10]. C57BL/6 and CBA mouse strains succumb to PbA-induced cerebral malaria, whereas others such as DBA and C58, are resistant to experimental cerebral malaria (ECM), dying instead of hyper-parasitemia and anemia [10,63]. The findings here showed that gerbils survived longer (11-19 days) than C57BL/6 and CBA mice (6-10 days), challenged with the same concentrations (1 × 10^6^ pRBC) of PbA [10,55,64]. However, the survival of gerbils following PbA infection was similar to that of DBA and C58 mice, which survived for 11-18 days and 15-22 days post-infection (pi) [10], respectively.

The high mortality rate of PbA infection in gerbils can be attributed to high parasitemia and anemia, which have been implicated in other non-ECM mouse models [55,63]. High parasitemia of above 40% pRBC observed in this study is in line with high parasitemia (> 60% pRBC) reported previously in non-ECM mouse models [2,55], whereas parasitemia < 20% pRBC has been reported for ECM-susceptible mice [60,63]. The low parasitemia observed in ECM mice has been described to be due to sequestration of PbA in organs such as the brain, spleen, liver and lungs, which lead to a reduced presence of the parasites in the peripheral blood [60].

Clinical symptoms such as weight loss and hypothermia were monitored. Hypothermia, defined as having a temperature below 30 °C, has been associated with hemorrhage in the brain and early death in ECM-susceptible mice [18]. In addition to the suggested association between hypothermia and ECM, hypothermia below 36 °C has also been identified as a marker for terminally ill rodents in an infectious bacterial disease model [43]. In a *P. chabaudi chabaudi* (AS) infection, hypothermia has been found to correlate with the parasitemia level, where resistant or resolving (B10 knockout) mice showed no hypothermia with peak parasitemia 20–30%, compared to susceptible (DBA/2 and A/J) mice, with peak parasitemia 50–60% [17]. The current findings in gerbils with hypothermia (below 35 °C) are similar to those reported by Bopp *et al.* [10], in both mice susceptible and resistant to CM induced by PbA. Additionally, hypothermia in rats has been associated with increased turnover of 5-hydroxytryptamine (5-HT serotonin) in the brain, which is a putative neurotransmitter, leading to both lower food intake and lower body temperature [19].

Overall, the data do not support the conclusion that gerbils die as a result of CM. This is due to the fact that none of the gerbils showed neurological symptoms which include ataxia, convulsion and deviation of the head. A report by Amani *et al*. [2] had shown that the genetic background of the mouse affects the disease outcome of PbA infections, but also that the cloned lines of PbA differ in their ability to induce ECM. As the PbA in this study was adapted to gerbils prior to the experiments, it is possible that the ability of PbA to induce ECM was modulated.

Severe malaria anemia (SMA) has been identified as one of the causes of mortality in ECM-resistant mouse models [55]. Findings here showed that gerbils suffered from severe anemia with significant low hemoglobin (Hb) concentrations (< 3 g/dL) and total RBC counts (< 9 × 10^8^ RBC/mL) during the course of infection. According to the World Health Organization (WHO), the standard measurement for SMA is hemoglobin (Hb) concentration < 50 g/L or 5 g/dL [75]. The hematocrit shown here is similar to the low hematocrit (< 10% PCV) observed in C57BL/6 mice infected with PbA and PK173 [53]. Also, Hb and total RBC count of < 40 mg/mL and 20 × 10^8^ RBC/mL, respectively have been reported during PbA infection in wild-type and knockout mice [3]. The underlying mechanisms of the factors contributing to severe malaria anemia (SMA), which includes dyserythropoiesis, phagocytosis of infected and uninfected RBCs, and erythrocytic suppression, are still poorly understood [46]. Helegbe *et al.* [38] have suggested that auto-antibodies play a potential role in the destruction of uninfected RBC in semi-immune mice. Host genetic factors may also influence the outcome of auto-immune-mediated mechanisms in RBC destruction [38,46]. Also, *Plasmodium* by-products, mainly hemozoin, have been suggested as a contributing factor for suppressed erythropoiesis, low reticulocytosis, and malaria anemia, by inhibiting the proliferation of erythroid precursors [71]. It has been proposed that SMA is mediated partly by immune-pathogenic mechanisms, mostly through a hyper-activated phagocytic system, which thus aids the destruction of uninfected RBCs [29]. However, more molecular evidence is still required to determine the major cause of severe anemia in *Plasmodium* infections.

The role of innate immunity as a protective response to malaria infection has been established [62,69,77]. Also, overproduction of cytokines has been implicated in the pathogenesis of severe malaria [5,48,65]. Previous reports have shown that malaria infection is associated with the development of Th1 cytokine response such as IL-1, IL-6, IFN-γ and TNF-α [40,45]. These studies are in agreement with the present findings, with gerbils eliciting pro-inflammatory cytokines in response to PbA. This study shows that gerbils did not respond early to PbA infection as revealed by expressions of inflammatory cytokines such as IFN-γ and TNF (Figure 6). Early production of IFN-γ has been suggested to correlate with protection from lethality of *P. yoelii* infection [40,45]. Moreover, late production of IFN-γ has also been suggested to be crucial in the development of CM [34]. Inflammatory cytokines such as tumour necrosis factor- α (TNF-α), interleukin-1 (IL-1) and IL-6, have been described to correlate with severe malaria but the major role of TNF-α has been linked with parasite killing [5,44]. Interferon gamma (IFN-γ) on the other hand, has been identified to be associated with pathogenesis and protection against CM [3], as well as controlling blood stage *Plasmodium chabaudi AS* [70]. Nonetheless, our study shows that both IFN-γ and TNF are persistently elevated for over 8 days during the 11-day time course. Hence, it can be suggested that the persistent elevation of innate immune response such as Th1 cytokines by gerbils is part of the host's immune response to eliminate the parasite.

Although elevated levels of serum IL-6 have been reported in ECM-susceptible mice [60], its role in the severity of the disease in mice is yet to be ascertained. However, in clinical studies, IL-6 has been found to be associated with hyper-parasitemia and human CM [20,49,74]. The present study shows IL-6 mRNA to be the least elevated in all the inflammatory cytokines measured in the plasma. This shows there is still a need to study the role of IL-6 in severe malaria.

It has been suggested that IL-10 plays an important role in immune regulation by down-regulating pro-inflammatory cytokines (such as TNF, IL-6 and IL-12), thereby inhibiting Th1 function and activities of natural killer cells [11,49]. This is further supported by a study by Li *et al*. [47] on the pathology of *Plasmodium chabaudi chabaudi* in C57BL/6 mice, showing regulatory cytokines such as transformation growth factor beta (TGF-β) and IL-10 to be crucial in modulating the magnitude of immunopathology during malaria infection. More so, other studies have suggested that the balance of anti-inflammatory to pro-inflammatory cytokines produced during *Plasmodium* infection determines the severity of malaria outcome [6,21]. However, more evidence is still required to determine whether the ratio or regulation of anti-inflammatory to pro-inflammatory cytokines would lead to protection or exacerbation of the host towards severe malaria.

Here, this study demonstrated that the gerbils respond to PbA infection by eliciting a combination of Th1 and Th2 cytokine responses. Immuno-regulatory cytokine such as IL-10 was significantly elevated in the three organs tested during the course of infection. This might explain the reason gerbils were resistant to induced CM by PbA. Also, the severity of anemia has been suggested to be dependent on levels of TNF-α relative to anti-inflammatory cytokine IL-10 [46]. This has also been observed in clinical studies where a low ratio of plasma TGF-β and IL-10 to TNF-α was associated with severe malaria anemia in young children in malaria endemic communities in Africa [1,57].

Identification of malaria parasites in formalin fixed paraffin embedded (FFPE) tissues has always been subject to many different methods and interpretations. Results from routine hematoxylin and eosin staining require highly skilled microscopists and mostly rely on the malaria parasite's visible pigments (hemozoin) which can easily be confused with deposits or pigments from formalin or other tissue processing reagents. However, in the present study, a *Plasmodium* genus DNA probe was employed to detect the presence of PbA iRBC in different tissues. Surprisingly, the findings show PbA to be present in tissues such as the brain, liver, lungs, kidneys and spleen, and this can be visualized under lowest magnification with the aid of an *in situ* hybridization method. Previously, a chromogenic *in situ* hybridization method had proven to be a sensitive and specific tool for detection of *Plasmodium* parasites in FFPE tissues [26,27,41]. However, these studies were so far only conducted on avian malaria parasites, and with this study, it can be recommended that this highly powerful tool could be adopted in a human clinical setting.

Furthermore, sequestrations of malaria parasites in the tissues are considered to be critical for disease pathogenesis [28,60]. The absence of mature trophozoites and schizonts of *P. falciparum* in human peripheral blood circulation has been suggested as evidence for sequestration of these stages [30]. Conversely, only the schizont stage of PbA has been found to sequester, while the matured trophozoites and all stages of gametocytes remain in circulation [54].

Previous works have shown that accumulation of PbA-infected red blood cells (iRBCs) can be found in organs such as the brain, liver, lungs, spleen, kidneys and adipose tissues in different murine models [4,31,51,55]. These reports are similar to the present findings with gerbils, where PbA iRBCs were also found to accumulate in the brain, liver, lungs, kidneys and spleen. Some researchers have suggested that the sequestration of *P. berghei* in the brain is not associated with ECM [13,31], while some suggest otherwise [4,37]. Although this model shows the presence of PbA iRBCs in the brain, interestingly, no clinical symptoms of neurological effects were observed.

## Conclusion

This study shows gerbils to be susceptible to PbA infection with pathological symptoms such as weight loss, hypothermia, anemia, splenomegaly and hepatomegaly. In addition, gerbil immune response to PbA showed the production of both pro-inflammatory cytokines (IFN-γ and TNF) and an immune-modulatory cytokine (IL-10). More importantly, we speculate PbA to have sequestered in the organs, as observed in *in situ* hybridization and H & E staining. Overall, the findings support the use of gerbils as an experimental model for severe malaria, although its limitations include the lack of gene knockout gerbils to further explore the roles of the cytokines in this study. Also, there is a constraint in the ability to quantify gerbil cytokine protein levels, as there are no kits available commercially. There is still a need to further understand the role of accumulated PbA in infected tissues of the host.

## Authors' contributions

Q.O.J., L.T.K., R.M., K.T.W. and I.V. designed and developed the research study; Q.O.J. and L.T.K., performed experiments, analyzed and discussed data, and wrote the paper. R.M., K.T.W. and I.V. reviewed and discussed the experimental data, provided materials and wrote the paper. K.C.O. and Y.L.L. provided the ISH probe, performed the ISH and histology experiments. P.U.B., J.W.K.L. and S.S. performed experiments and reviewed and discussed the experimental data. All authors read and approved the final manuscript.

## Conflicts of interest

The authors declare that they have no conflicts of interest in relation to this article.
